# Role of Polyanions and Surfactant Head Group in the Formation of Polymer–Colloid Nanocontainers

**DOI:** 10.3390/nano13061072

**Published:** 2023-03-16

**Authors:** Elmira A. Vasilieva, Darya A. Kuznetsova, Farida G. Valeeva, Denis M. Kuznetsov, Lucia Ya. Zakharova

**Affiliations:** Arbuzov Institute of Organic and Physical Chemistry, FRC Kazan Scientific Center, Russian Academy of Sciences, Arbuzov Str. 8, 420088 Kazan, Russia

**Keywords:** polymer–colloid complex, surfactant, polyelectrolyte, human serum albumin, polyacrylic acid, critical aggregation concentration

## Abstract

Objectives. This study was aimed at the investigation of the supramolecular systems based on cationic surfactants bearing cyclic head groups (imidazolium and pyrrolidinium) and polyanions (polyacrylic acid (PAA) and human serum albumin (HSA)), and factors governing their structural behavior to create functional nanosystems with controlled properties. Research hypothesis. Mixed surfactant–PE complexes based on oppositely charged species are characterized by multifactor behavior strongly affected by the nature of both components. It was expected that the transition from a single surfactant solution to an admixture with PE might provide synergetic effects on structural characteristics and functional activity. To test this assumption, the concentration thresholds of aggregation, dimensional and charge characteristics, and solubilization capacity of amphiphiles in the presence of PEs have been determined by tensiometry, fluorescence and UV-visible spectroscopy, and dynamic/electrophoretic light scattering. Results. The formation of mixed surfactant–PAA aggregates with a hydrodynamic diameter of up to 190 nm has been shown. Polyanion additives led to a decrease in the critical micelle concentration of surfactants by two orders of magnitude (from 1 mM to 0.01 mM). A gradual increase in the zeta potential of HSA–surfactant systems from negative to positive value indicates that the electrostatic mechanism contributes to the binding of components. Additionally, 3D and conventional fluorescence spectroscopy showed that imidazolium surfactant had little effect on HSA conformation, and component binding occurs due to hydrogen bonding and Van der Waals interactions through the tryptophan amino acid residue of the protein. Surfactant–polyanion nanostructures improve the solubility of lipophilic medicines such as Warfarin, Amphotericin B, and Meloxicam. Perspectives. Surfactant–PE composition demonstrated beneficial solubilization activity and can be recommended for the construction of nanocontainers for hydrophobic drugs, with their efficacy tuned by the variation in surfactant head group and the nature of polyanions.

## 1. Introduction

The systems based on oppositely charged surfactants and polyelectrolytes (PEs) are successfully used in the production of cosmetics, household chemicals, and drug delivery [1,2,3]. This is due to the fact that PEs can regulate surfactant functional properties, such as adsorption at the water–air interface, self-assembly in the bulk solution, rheology, and solubilization effect; the visa verse characteristics of polymer can be purposefully modified by surfactants added [4,5,6]. Therefore, the formation of mixed polymer–colloid systems and evaluation of factors controlling their properties may be considered an effective and soft tool for the construction of functional materials and tailoring their activity. This encouraged us to focus on such kinds of systems, with special attention paid to cationic surfactants and PE of both synthetic and natural sources.

Interactions between surfactants and PEs in the bulk solution and at the water–air interface have been systematically studied by many research groups, including ours [4,7,8,9,10]. Meanwhile, these systems continue to attract the attention of researchers, with the variety of self-assembling nanostructures such as micelles, vesicles, polyelectrolyte capsules, nanolayers, nano- and microparticles involved [11,12,13,14]. This research activity emphasizes the urgency of the study of surfactant–PE systems, which is based on the following reasons:

(i) The formation of surfactant–PE complexes is contributed by a variety of noncovalent intermolecular interactions, including hydrophobic effect, hydrogen bonds, Van der Waals, and electrostatic forces. These interactions depend on various factors, such as the structure of components, e.g., chain rigidity, charge density, the molecular weight of PE, the nature of the head group, and hydrophobicity of surfactants [8,15,16,17], which is responsible for the high specificity and versatility of aggregation characteristics of the systems. Moreover, different values of critical concentrations corresponding to the onset of mixed aggregation may be obtained depending on the methods used [18,19]. Such multifaceted structural behavior is supported by a number of publications focusing on mixed systems upon the variation of the structure of components or solution conditions [20,21]. Importantly, in the case of oppositely charged surfactant–PE mixtures, an extended region of precipitate usually occurs upon charge neutrality, while soluble complexes are formed in the area of an excess surfactant or PE concentration [15]. It was found that in mixed PAA–surfactant systems, the region of heterogeneity is regulated by the hydrocarbon tail length of the amphiphiles [22]. As shown in work [19], the region of turbidity occurring close to zero zeta potential strongly depends on the surfactant–PE ratio and tends to shift toward the higher surfactant concentrations upon the increase in fixed PE concentration. Kogej et al. emphasized that even a slight change in the structure of a polyion significantly affects the binding of surfactants to the PE [20,21];
(ii) It is noteworthy that for mixed polymer–surfactant systems, different models were developed for the description of their structural behavior, e.g., the so-called necklace model [23]. Even though the use of this model in the system based on oppositely charged components is documented [24], this approach is not general. In contrast to surfactant–nonionic polymer systems, the nature of the surfactant head group has a significant effect on the surfactant–PE interaction [18,25]. This can be exemplified by PAA–surfactant systems involving two cationic surfactants with morpholinium and triphenylphosphonium head groups. The former systems demonstrated strong synergetic behavior, with a marked decrease (up to two orders of magnitudes) in the aggregation threshold. On the contrary, only a slight, if any, effect was observed in the latter system. Similarly, the nature of PE may significantly affect the solution behavior of mixed surfactant–PE complexes [26]. Unlike with DABCO-based surfactant and poly(sodium 4-styrenesulfonate) system demonstrating one breakpoint on the surface tension isotherms and an increase in critical concentrations, two breakpoints and pronounced synergetic behavior (decrease in critical aggregation concentration compared to single surfactant) occurred in the case of PAA–surfactant system.
  Taking into account these considerations, it should be stressed that unlike with surfactant–polymer systems based on uncharged polymers, complicated structural behavior controlled by a variety of factors is typical for the surfactant–PE interactions, especially involving oppositely charged components, with no uniform conception occurring so far. This demonstrates the necessity of additional studies to elucidate the mechanisms responsible for their behavior and develop the optimal model for describing and predicting the interactions and physicochemical properties in such pairs;
(iii) Structural behavior of supramolecular systems contributed by noncovalent interactions tends to demonstrate stimuli responsibility. In this respect, surfactant–PE systems based on unbuffered PAA are of particular interest due to the fact that they demonstrate pH-dependent behavior and can be controlled by changes in solution conditions. Therefore, the information on the self-assembling, morphological, and functional activity of such compositions is of practical importance;
(iv) Since many innovative drugs are lipophilic, great efforts focused on the development of nanosized carriers to increase their solubility and biocompatibility. surfactant–PE nanostructures may be of interest as carriers due to the ease of their formation. According to the literature data, including our studies, mixed systems based on amphiphiles and PE can be used as nanocontainers for the delivery of hydrophobic substances, specifically medicines [3,11,12,13,27,28];
(v) Polymer–surfactant complexes are biomimetic systems, and their study makes it possible to simulate the interaction of charged amphiphiles with natural biopolymers (nucleic acids, proteins, polysaccharides) and lipids [29,30], factors of enzyme catalysis [31], etc. Moreover, they may be considered the simplest models for studying membrane–drug interactions (e.g., antimicrobial preparations exert their effects by interacting with biological membranes) [32]. Essentially, the self-aggregation study of mixed surfactant–PE compositions is necessary for a comprehensive description of their solution behavior and functional activity. The knowledge gained can provide a better understanding of optimizing the composition and properties of nanocontainers based on surfactants and PEs;
(vi) The systems based on surfactant and natural PE are of great importance, e.g., the interaction of cationic surfactants with globular proteins-albumins-is widely studied due to the bioapplication perspective [33,34,35]. Albumins are blood transport proteins and have unique properties (commercial availability, water solubility, biodegradability, biogenicity, and ability to accumulate in tumor tissues), which makes them promising candidates for the development of protein-based nanocontainers [36,37]. Complexation involving cationic surfactant–protein systems is of considerable interest since the interaction of proteins with amphiphilic molecules can lead to compaction or unfolding of the proteins, which is an important property for medicine and pharmacology [33];
(vii) Human serum albumin (HSA) is one of the most important plasma proteins. It consists of 585 amino acid residues with three α-helical domains, stabilized by disulfide bonds [36,38]. Importantly, HSA is non-toxic, has a high protein–drug binding ability, and is widely used to transport exogenous and endogenous ligands [39,40]. Currently, HSA is conjugated to numerous drugs such as insulin, paclitaxel, interferon, doxorubicin, etc. [41,42]. As shown in [43,44], the use of mixed surfactant–protein systems leads to a synergistic effect in self-assembly behavior and solubility of lipophilic compounds and, consequently, improves their biocompatibility. Meanwhile, few publications are available on the self-assembly and morphological behavior of the HSA–surfactant systems [45,46,47], which provide evidence of the deficiency of fundamental information in this field and highlight the novelty of the data expected.

**Figure 1:**
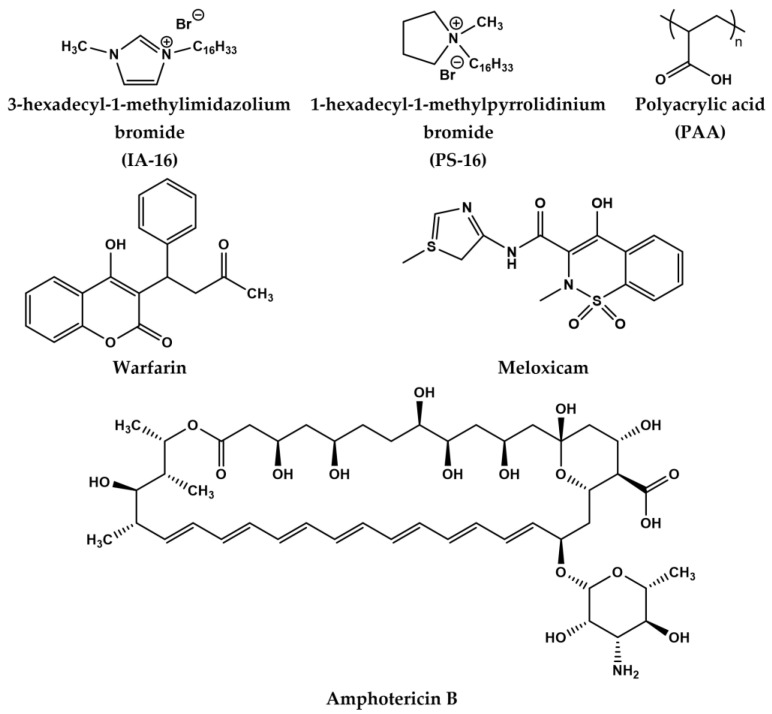
Structural formulas of surfactants, PAA, and drugs.

Based on these reasons, PAA and HSA were chosen as synthetic and natural PE for the study of their mixed behavior with cationic surfactants, which aims at elucidating the factors controlling physicochemical characteristics, functionality, and practical potential of such compositions. At the first stage, the complexation between cationic surfactants with a different head group (imidazolium (IA-16) and pyrrolidinium (PS-16)) with (polyacrylic acid and human serum albumin) is studied, with the effect of PAA and HSA on the aggregation and size characteristics of amphiphiles evaluated. The mechanism of the protein–surfactant interaction in an aqueous solution is studied with subsequent calculation of quantitative parameters of component binding: Stern–Volmer (K_SV_) and binding (K_a_) constants, the number of binding sites (n), thermodynamic parameters of the interaction of components (changes in enthalpy ΔH^0^, entropy ΔS^0^, and Gibbs free energy ΔG^0^) are estimated. In the second stage, the possibility of using the polymer–colloid complexes for the solubilization of lipophilic drugs of various types (warfarin, amphotericin B, meloxicam) was assessed. The structural formulas of the compounds used are shown in Figure 1.

## 2. Materials and Methods

### 2.1. Materials

Polyelectrolytes (PAA, 1800 g/mol, ≥99%), HSA (66 kDa)); fluorescence probe (pyrene, ≥99%), and drugs (warfarin (≥98.0%), meloxicam, amphotericin B) were purchased from Sigma–Aldrich (St. Luis, MO, USA) and used as received. The PS-16 and IA-16 were synthesized similarly to the procedure indicated in refs. [48,49], respectively. 

### 2.2. Sample Preparation

Milli-Q system (Millipore S.A.S. 67120 Molsheim, France) was used for the purification of the solvent (18.2 MΩ·m resistivity) for the preparation of samples. Ultra-purified water was applied as a solvent for the preparation of amphiphile-PAA and amphiphile-HSA mixtures. All solutions (except stock ones) were prepared using a volume dilution method. The concentration of HSA in all binary surfactant–HSA systems was constant and amounted to 5 µM [47]. Meanwhile, in surfactant–PAA systems, the PE concentrations were 1, 3, and 5 mM.

### 2.3. Surface Tension Measurements 

The surface tension of surfactant–HSA and surfactant–PAA mixtures was measured using a Krűss K6 tensiometer (KRUSS GmbH, Hamburg, Germany) via the Du Nouy ring detachment method at 25 °C. Triplicate reproducible surface tension values with a deviation of no more than ±0.1 mN/m were taken into account.

### 2.4. The Dynamic and Electrophoretic Light Scattering

The dynamic and electrophoretic light scattering (DLS and ELS, Zetasizer Nano, Malvern Instruments Ltd., Worcestershire, UK) methods were used to measure the hydrodynamic diameter (D_h_), zeta potential (ζ), and the polydispersity index of aggregates formed based on surfactants and Pes. The concentration of samples was between the critical aggregation concentration-1 (CAC-1) and CAC-2 regions. Measurements were taken in triplicate at 25 °C using a disposable folded capillary cell. D_h_ and ζ were obtained using Stokes–Einstein and Helmholtz–Smoluchowski equations, respectively [50]. PdI was calculated using cumulative second-order correlation function analysis.

### 2.5. Fluorescence Spectroscopy 

The fluorescence spectra of pyrene (in the concentration of 1 μM) in amphiphile/PAA aqueous solutions were recorded in the range of 350–500 nm using fluorescence spectrophotometer Hitachi F-7100 (Hitachi High-Tech Corporation, Tokyo, Japan) at 25 °C. The excitation wavelength was 335 nm, and the scanning speed was 120 nm/min. The fluorescence intensity of the I peak at 373 nm and the III peak at 384 nm was determined by the spectra obtained. 

Registration of fluorescence spectra of amphiphile–HSA mixed systems was performed at 25 °C, 30 °C, 35 °C, 40 °C, and 280 nm excitation wavelength. Emission spectra were recorded in the range of 290–450 nm with 240 nm/min scan speed. All measurements were conducted in a 1 cm cuvette. 

Based on the obtained fluorescence spectra, the Stern–Volmer constants for various temperatures were calculated using the following equation [51]:

(1) $$\frac{F_{0}}{F}=1+K_{SV}\left[Q\right]=1+k_{q}\cdot \tau_{0}\left[Q\right],$$

where *F*_0_ and *F* are the fluorescence intensities in the absence and in the presence of the quencher (surfactant), respectively; *K_SV_* is the Stern–Volmer constant; [Q] is the concentration of surfactant; *k_q_* is the bimolecular rate constant of quenching; *τ*_0_ is an average lifetime of fluorophore (HSA) in the excited state in the absence of quencher, which equals 10^−8^ s [51].

The bimolecular rate constant of quenching *k_q_* was evaluated using Equation (2):

(2) $$k_{q}=\frac{K_{SV}}{\tau_{0}}$$

Determination of binding constants of components *K_a_*, the number of binding sites *n,* and thermodynamic characteristics of surfactant–HSA systems was performed on the basis of the primary fluorescent data by Equations (3)–(5) [34]:

(3) $$lg\frac{F_{0}-F}{F}=lgK_{a}+nlg[Q]$$

(4) $$lnK_{a}=-\frac{\Delta H^{0}}{RT}+\frac{\Delta S^{0}}{R}$$

ΔG^0^ = ΔH^0^ − TΔS^0,^ (5)

where Δ*H*^0^ is the variation of standard enthalpy; Δ*S*^0^ is the variation of standard entropy, *R* is a universal gas constant (8.314 J·mol^−1^·K^−1^); *T* is absolute temperature; Δ*G*^0^ is the change of standard Gibbs free energy.

Registration of three-dimensional fluorescence spectra was performed in the range of excitation wavelength 200–350 nm with increments of 10 nm and emission wavelengths of 200–450 nm with increments of 10 nm [47]. The scanning speed was 240 nm/min.

### 2.6. Spectrophotometry

Absorption spectra of the polymer–colloid solutions were recorded using Specord 250 PLUS spectrophotometer (Analytik Jena AG, Jena, Germany). The solubilizing ability of the surfactant-HSA and surfactant-PAA systems was determined by adding an excess of the different drugs (Warfarin, Amphotericin B, and Meloxicam). The absorption spectra of solutes were registered in the range of 200–450 after 24 h at room temperature. Measurements were taken in triplicate at 25 °C using a 0.5 cm quartz cuvette. Primary spectral data were used for extraction of the maximum optical density values at λ = 305 nm (extinction coefficient of Warfarin equals 17,400 M^−1^·cm^−1^) for Warfarin, at λ = 385 nm (extinction coefficient of Amphotericin B equals 53,935 M^−1^·cm^−1^) for Amphotericin [47], and at λ = 364 nm (extinction coefficient of Meloxicam equals to 14,610 M^−1^·cm^−1^) for Meloxicam. The solubilization capacity of complexes (*S*) was calculated from the concentration dependences of the optical density (D) using the following equation: S = b/ε, where b is the slope of the D/l = f (C) dependence, l is the cuvette thickness, C is the surfactant concentration, and ε is the extinction coefficient.

### 2.7. Transmission Electron Microscopy (TEM)

TEM images were obtained at the Interdisciplinary Center for Analytical Microscopy of Kazan (Volga Region) Federal University with a Hitachi HT7700 Exalens microscope (Hitachi, Tokyo, Japan). The images were acquired at an accelerating voltage of 100 kV. Samples were dispersed on 300 mesh 3 mm copper grids (Ted Pella) with continuous carbon–formvar support films.

### 2.8. Data Analysis and Statistics

All measurements were carried out at least three times. Data were performed via OriginPro 8.5 (OriginLab Corporation, Northampton, MA, USA) and expressed as mean values ± standard deviation (SD). The statistical significance of the difference between the values of the solubilization capacity and hydrodynamic diameter was determined using a one-way analysis of variance (ANOVA) test. Differences were considered significant at a *p*-value < 0.05. 

## 3. Results and Discussion

### 3.1. Surfactant–PAA Nanocarriers 

#### 3.1.1. Phase Behavior of the Surfactant–PAA Systems

Our early investigations demonstrated the complexation of cationic surfactants with pyrrolidinium and imidazolium head groups containing a hydroxyethyl moiety with PAA [52,53]. It was shown that the properties of polymer–colloid systems significantly depend on many factors, including the surfactant head group structure [18] and the presence of functional groups. It was of interest to reveal new structure–property relationships, which may allow elucidating additional factors to control the functional activity of the systems and markedly widen their practical application. Typically, the appearance of turbidity and even the formation of insoluble precipitate occur in a certain concentration range in polymer–colloid solutions. It was shown that the addition of electrolytes could prevent phase separation [54]; however, they significantly reduce the critical micelle concentration (CMC) of cationic surfactants. Therefore, before an investigation of the adsorption of surfactant–PE systems at the water–air interface, it is useful to study the bulk phase behavior of these systems. 

**Figure 2:**
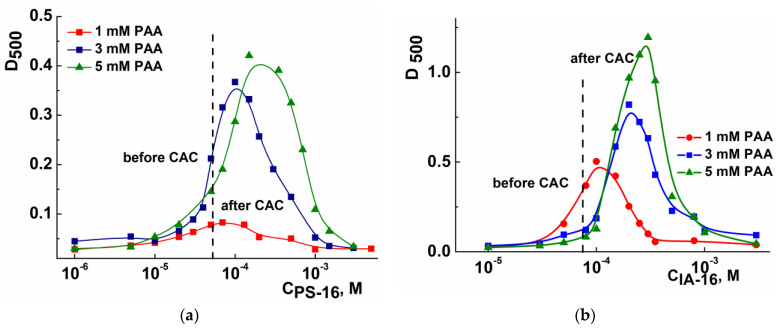
Absorbance of PAA-PS-16 (**a**) and PAA-IA-16 (**b**) systems at 500 nm under different surfactant concentrations and fixed concentrations of PAA; 25 °C.

Figure 2 shows the spectrophotometry data, which demonstrates the change in the absorbance (turbidity) of the mixed PAA-PS-16 (a) and PAA-IA-16 compositions (b) upon increasing surfactant concentration. Looking ahead, the critical aggregation concentrations (CAC) of surfactant–PAA systems are in the clear solutions region (absorbance up to 0.2). The turbidity increases above the CAC region (~0.05 mM), and close to the surfactant CMC (1 mM), the solutions become clear again. It is noteworthy that in the case of imidazolium surfactants, more cloudy solutions are formed, independently of the polymer concentration (absorbance 1.2), compared to systems with pyrrolidinium surfactant (absorbance 0.4). This may indirectly indicate that stronger electrostatic interactions occur between the components. Meanwhile, phase separation is not observed in either system. The phase behavior of oppositely charged surfactant and PE mixtures is closely related to their charge characteristics. Cloudy solutions are observed in the neutral charge area, with precipitation occurring in some cases, whereas soluble complexes are formed in the presence of a charge (with an excess of surfactant or PE) [15].

**Figure 3:**
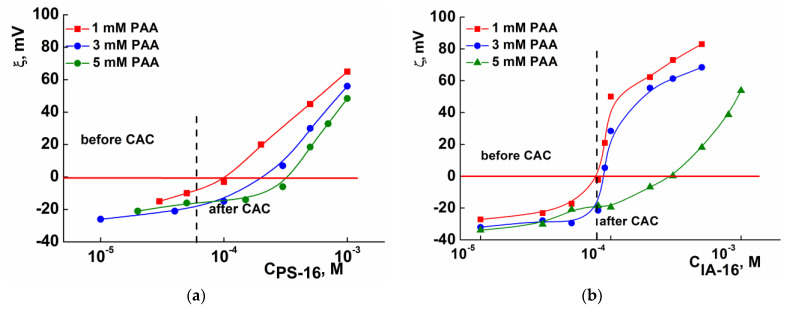
Zeta potential of PS-16-PAA (**a**) and IA-16-PAA (**b**) systems under different surfactant concentrations and fixed concentrations of PAA; 25 °C.

The charge of the surfactant–PE complexes was estimated by measuring their zeta potential. Figure 3 presents electrophoretic light scattering data reflecting the dependence of the zeta potential of surfactant–PAA systems upon varying the components’ ratio. It is known that the presence of a charge prevents the aggregation of particles and determines the stability of colloidal systems [55]. Electrostatic stabilization requires the magnitude of zeta potential of about 30 mV [56]. Gradual increase in the zeta potential from −25 mV to +80 mV, depending on the ratio of the components, was shown for both systems. It should be noted that co-aggregation in the bulk solution always begins in the negatively charged region, i.e., with an excess of the polyanion. Despite the low charge density of PAA (ionization degree is around 8% at pH = 4 [19]), a rather significant zeta potential (Figure 3) indicates that the electrostatic forces are involved in the interaction between the components. At the same time, the hydrophobic effect and hydrogen bonding can also play an important role in the surfactant complexation with PAA. In the case of weak PEs, the presence of a charge depends on the solution pH. In this study, spontaneous solution pH of ca.4 was maintained. According to Manning’s theory [57], at a low degree of charge density of the PE chain, the binding of the surfactant to PAA is mainly driven by the hydrophobic effect. Precipitation is assumed to result from hydrogen bonding that occurs between uncharged segments of the PAA chain. At a high charge density of PAA, hydrophobic binding weakens due to the increase in the charged character of PE chains and their binding to the surfactant via electrostatic interactions.

#### 3.1.2. Self-Assembly of the Oppositely Charged Surfactant–PAA Systems

Critical aggregation concentration (CAC) of cationic surfactant–anionic PE mixed systems can be several orders of magnitude lower than the surfactant CMC. This is due to the fact that the addition of the anionic PE significantly reduces the electrostatic repulsion force between the head groups of the cationic surfactant. However, as mentioned above, in the case of weak PE, low CAC values can be caused by not only electrostatic interactions but also by hydrophobic effect and hydrogen bonding. Amphiphiles can bind to PE in both monomeric and micellar forms depending on the concentration. In this study, the critical concentrations were determined by the Du Nouy ring method (tensiometry). Primarily, the tensiometry reflects the surfactant–PE system behavior at the water–air interface and only then by their aggregation properties in the bulk solution. Furthermore, the surface tension curves of the oppositely charged surfactant–PE systems can be more complicated depending on the components ratio and have several breakpoints. In our work, a variety of methods are always involved in determining CAC/CMC. 

**Figure 4:**
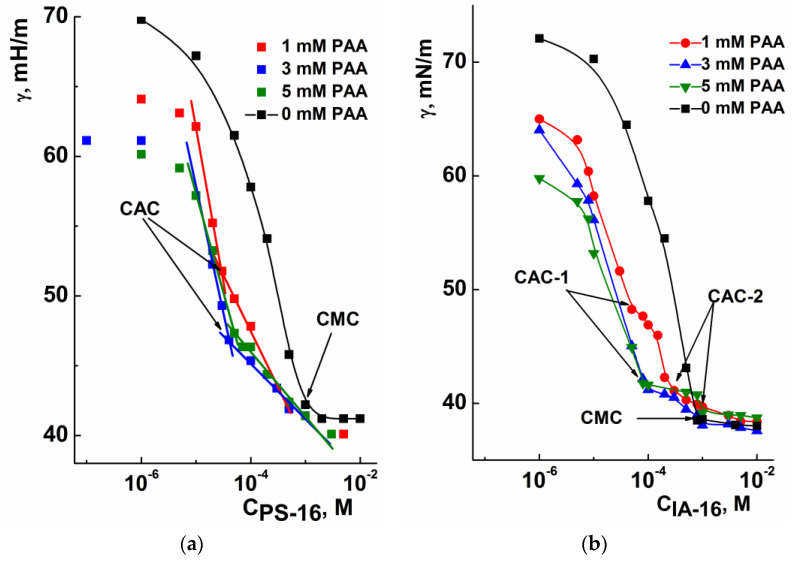
Surface tension isotherms of mixed PS-16-PAA (**a**) and IA-16-PAA (**b**) systems under different surfactant concentrations and fixed PAA additives; 25 °C (CAC and CAC-1 have the same meaning).

Figure 4 shows the surface tension isotherms of the PS-16 (a) and IA-16 (b) as a function of the surfactant concentration in the presence of PAA. The PAA-IA-16 concentration curves demonstrate a two-step aggregation, which is typical for oppositely charged surfactant–PE mixtures [58]. The first breakpoint in Figure 4b is ascribed to CAC-1 and characterizes the concentration of the beginning of complexation in the bulk solution. At the second one (CAC-2), a saturation of the PE chain by surfactant aggregates and the formation of pure micelles occurs. In the case of the pyrrolidinium surfactant (Figure 4a), only one breakpoint is clearly visible (CAC) in the surface tension isotherms, and curves do not reach a plateau. Taylor et al. reported that PE additives could initiate the adsorption of surfactant at water–air interfaces in both monomeric and micellar forms, causing a decrease in the surface tension after reaching CAC [58]. As can be seen from Table 1, the CAC-1 of mixed surfactant–PAA systems is 10–15 times lower than the CMC of pure surfactants, independently of their head group. An increase in the concentration of PAA has little effect on the CAC values. It should be noted that the surface tension of mixed systems is much lower compared to pure surfactant up to the aggregation thresholds. This is probably due to the surface activity of the PE, which was shown earlier [19].

An additional highly sensitive method of fluorescence spectroscopy using pyrene was engaged to confirm the tensiometric measurements. This method is widely used for investigation not only of pure micellar solutions but also of mixed systems based on oppositely charged polymers and surfactants [59]. Furthermore, both methods are well-complemented since tensiometry primarily provides information on the water–air interface, while fluorescence spectroscopy describes bulk aggregation. The pyrene properties depend on the polarity of its microenvironment, and, therefore, the fluorescence spectra provide information about fluorophore location. The polarity index (I_1_/I_3_) is used as a measure of polarity and is defined as ratios of the first (373 nm) and third (384 nm) peaks in the pyrene spectrum. The decrease in the polarity index indicates the decrease in the polarity of the pyrene microenvironment caused by its solubilization in the hydrophobic core of the micelle. The determination of the CMC of surfactant is based on this property. 

**Figure 5:**
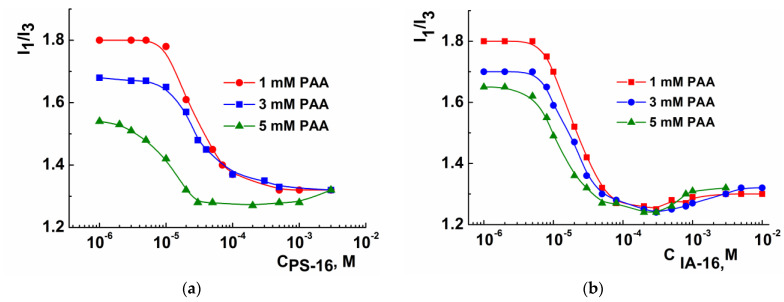
Polarity index (I_1_/I_3_) as a function of surfactant concentration in the PS-16-PAA (**a**) and IA-16-PAA (**b**) mixtures under different PAA concentrations; 25 °C.

Figure 5 demonstrates the I_1_/I_3_ plots as a function of amphiphiles concentration in the mixed PAA-PS-16 (a) and PAA-IA-16 (b) systems upon various PAA concentrations. In both cases, the addition of PE leads to a decrease in the CMC by two orders of magnitude (from 1 mM to 0.01 mM). The aggregation thresholds of mixtures are slightly depend on the PAA concentration. The CAC values were determined by fluorimetry, which was lower than tensiometry data (Table 1). Discrepancy in results may be due to different sensitivities of the methods and sample preparation. It should be noted that the polarity index noticeably decreases with increasing in PAA concentration. This is consistent with work reporting on the sensitivity of the pyrene microenvironment to the chitosan concentration in the mixed systems [60]. This indicates that the presence of a macromolecule induces the formation of more densely packed micellar aggregates due to a decrease in the electrostatic repulsion of amphiphile head groups. In the case of an imidazolium surfactant (Figure 5b), the concentration dependence of the polarity index has a minimum and begins to rise in the CMC region, followed by a plateau. This is probably due to the morphological transitions and indicates the formation of pure micelles.

#### 3.1.3. Dimensional Characteristics and Morphology of Surfactant–PAA Systems

The hydrodynamic diameter (D_h_) of the oppositely charged surfactant–PE systems under the different molar component ratios was determined by dynamic light scattering. D_h_ of the PS-16 and IA-16 micelles is around 2–5 nm at a concentration of 1 mM (CMC), which corresponds to spherical micelles. Single PAA solution at a concentration range of 1–5 mM contains small aggregates with D_h_~10 nm and much larger particles ~250 nm. Probably large aggregates are the combination of several hydrogen-bonded macromolecules, which are predominant at the low degree of the PAA ionization [19]. The D_h_, zeta-potential, and polydispersity index (PdI) in the CAC-1/CAC-2 region under excess of PE are summarized in Table 2. Since the CAC-1 values determined by tensiometry and fluorometry are slightly different, average concentrations were chosen to determine the size of the systems. One-way ANOVA testing demonstrated that there were statistical differences between D_h_ of mixed aggregates in both mixed compositions within a system at a constant concentration of PAA (Table S1). 

Aggregates with D_h_~90 ± 10 nm were detected in the CAC-1 region in the case of PAA-PS-16 mixtures with 1 mM PAA and 179 ± 3 nm with the increase in PAA content up to 5 mM. This behavior is in line with the increases in the number of PE chains in the solution. The size of mixed aggregates decreases to 37 ± 5 nm with increasing concentration of surfactants to 1 mM. The mechanism of binding PAA to classical cationic surfactant-bearing ammonium head group at a low degree of ionization (α < 0.3) is well-described in [57]. In the absence of surfactant, the PAA chain is low-polar and forms a compact structure, while binding of PAA to amphiphile unfolds the PE, thereby contributing to the formation of large complexes through hydrogen bonding. When the surfactant concentration reaches the CAC-2, the PAA–amphiphile complex dissociates, which leads to a decrease in particle size. It should be noted that a monomodal distribution of particle sizes with low PdI not exceeding 0.45 was observed in all cases, which is good enough for oppositely charged systems [61]. A similar trend was observed in the case of imidazolium surfactant: the D_h_ of complex decreases from 137 ± 5 to 52 ± 19 nm with the increase in amphiphile concentration. The particle size does not change for 2 months at room temperature (25 °C), which indicates the formation of stable mixed nanostructures. 

**Figure 6:**
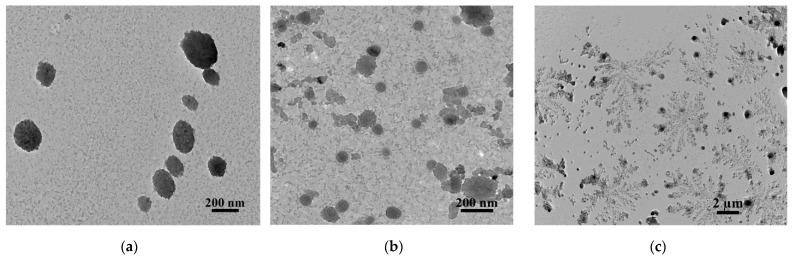
TEM-photo of PS-16-PAA structures at 0.05–1 mM, respectively, and different scales: 200 nm (**a**,**b**); and 2 μm (**c**).

Morphology of the mixed surfactant–PAA nanostructures was studied with transmission electron microscopy. Nanostructures based on pyrrolidinium surfactant and PAA close to tensiometric CAC (0.05 mM PS-16 and 1 mM PAA) were chosen for the visualization. TEM images are represented in Figure 6. In the presence of PAA, various types of particles with a rather high polydispersity are formed. Mostly spherical particles with a diameter from 80 to 200 nm are observed (Figure 6a,b). It should be noted that, in the case of DLS data, the formation of particles with D_h_ around 190 nm and low PdI is observed. The presence of highly aggregated particles or separate but smaller sizes is probably due to the removal of water during sample preparation. It is known that pearl-necklace-like aggregates can be formed in surfactant–polymer systems [24]. 

As can be seen from Figure 6a, some of the spherical particles are located in the same row without connection to each other. It was reported that the polymer chains between the beads are exposed to water, which provides low contrast when imaging the sample [62]. It should also be noted that the formation of spherical mixed PS-16-PAA aggregates may indicate the solubilization of the macromolecule in surfactant micelles, which contributes to an increase in the size of mixed systems compared to individual micelles (up to 5 nm for the PS-16 derivative [48]). 

Moreover, uniformly distributed large aggregates resembling dendrimers with a diameter of around 3 μm were observed (Figure 6c), similar to those previously shown for chitosan decorated liposomes [55]. However, in this case, the aggregates are 10 times larger than in the liposomal system. It should be noted that in this experiment, the surfactant–PAA aggregation was studied under large excess of PE, i.e., a low concentration of mixed aggregates takes place. Therefore, it is possible that large aggregates correspond to an unbound polymer, which, in turn, are aggregated with each other due to the presence of oppositely charged surfactants and the low degree of PAA ionization.

### 3.2. Surfactant–HSA Nanocarriers

#### 3.2.1. Self-Assembly of the Oppositely Charged Surfactant–HSA Systems

**Figure 7:**
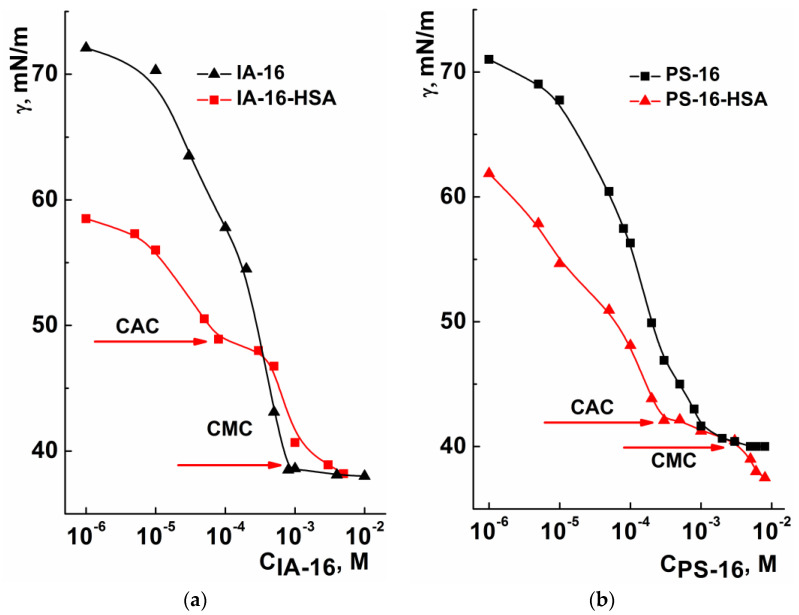
Surface tension isotherms for IA-16-HSA (**a**) and PS-16-HSA (**b**) systems as a function of surfactant concentration under constant HSA concentration (5 µM); 25 °C.

At the next stage of this work, the complexation of IA-16 and PS-16 with the natural polyanion–human serum albumin (HSA) was studied using a complex of physicochemical methods. The aggregation characteristics of the IA-16-HSA (Figure 7a) and PS-16-HSA (Figure 7b) systems were evaluated via tensiometry. The study of aggregation properties is important since it is known that surfactants can significantly affect the conformational behavior of proteins. This is due to the fact that the binding of proteins to surfactants causes a change in the intramolecular forces that maintain the secondary structure of the protein, causing conformational changes in the macromolecule [63]. However, the addition of proteins to surfactants can significantly change the properties of the adsorption layer and affect the surface tension of the amphiphile solution [44,63]. 

As can be seen from Figure 7, the surface tension of the mixed surfactant–HSA system is lower than that of pure surfactant, which is probably due to the surface activity of the protein [47,64]. Furthermore, two breakpoints were observed in the concentration dependences of the surface tension of mixed systems (0.08 mM and 1 mM for IA-16-HSA; 0.3 mM and 3 mM for PS-16-HSA). As mentioned above, the first breakpoint at low surfactant concentration is known as CAC-1. It corresponds to the saturation of the water–air interface with amphiphile molecules associated with the protein chain, as well as the beginning of the formation of surfactant–protein complexes [51,63]. The second breakpoint appearance, as in the case of synthetic surfactant–PE complexes, is probably associated with the saturation of HSA macromolecules with amphiphile molecules and corresponds to the formation of free micelles [52]. Interestingly, the formation of mixed IA-16-HSA complexes begins at a lower concentration of amphiphile (0.08 mM), unlike with PS-16-HSA systems (0.3 mM). As has been previously reported [35,63], the binding of the protein to amphiphile at the water–air interface is dominantly observed through electrostatic interactions at low concentrations of cationic surfactant. Hydrophobic interactions begin to predominate with the increase in surfactant concentration. Therefore, the difference in the complexation of the studied amphiphiles with HSA can be related to their different ability for electrostatic binding. Probably, electrostatic interactions are preferable for IA-16 due to the planar structure of the head group [49], compared to cyclic one in PS-16 with low conformational mobility [65].

**Figure 8:**
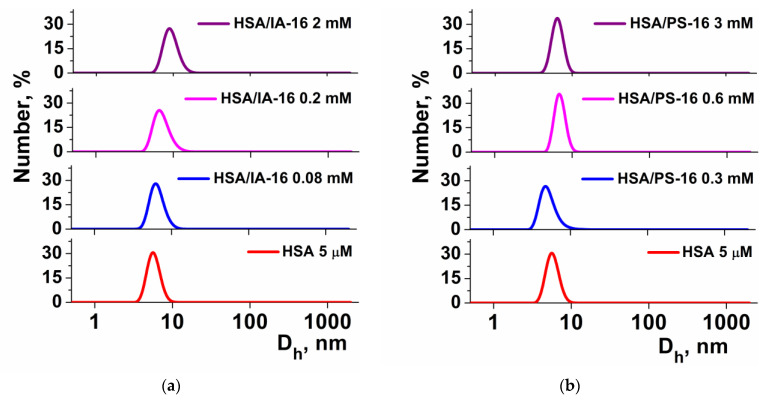
Number-averaged size distribution for mixed IA-16-HSA (**a**) and PS-16-HSA (**b**) systems; 25 °C.

As mentioned above, surfactant binding to HSA can cause various conformational changes in the protein that can affect its size. Therefore, the DLS method was used to determine the D_h_ of native HSA and mixed IA-16-HSA and PS-16-HSA associates. As can be seen from Figure 8, the D_h_ of pure HSA is in the range of 6–10 nm, which correlates to previously obtained data [47,66]. Different concentrations of cationic surfactants (IA-16 and PS-16) have a small effect on the size of the polypeptide. Both mixed complexes demonstrated similar sizes in the range of 4–10 nm. This is in agreement with minor changes in protein structure confirming specific binding since a significant increase in the size of the complexes would have indicated the loss of the tertiary and secondary polypeptide structure [67]. 

#### 3.2.2. Binding Mechanism Study between Surfactants and HSA

**Figure 9:**
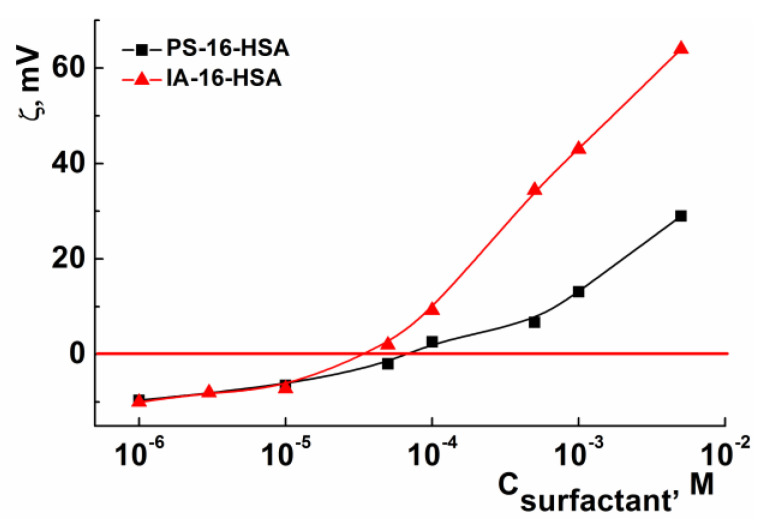
Electrokinetic potential (zeta potential) as a function of IA-16 and PS-16 concentration for mixed IA-16/HSA and PS-16/HSA systems (HSA concentration is 5 µM), 25 °C.

The zeta potential is a useful parameter to assess the surface charge of proteins. Changes in the HSA zeta potential can be the result of surface modifications of the macromolecule, unfolding/denaturation processes, conformational changes in the structure, and also electrostatic interaction in protein–ligand systems [68,69]. Therefore, the zeta potential of pure protein (at physiological pH = 7.4) and mixed IA-16-HSA and PS-16-HSA associates was evaluated by electrophoretic light scattering (Figure 9). The zeta potential of free HSA was ~−10 mV; cationic surfactant additives and an increase in their concentration led to a change of zeta potential from negative to a positive value. Such behavior in mixed systems testifies to the formation of protein–ligand complexes due to the electrostatic interaction [69,70]. Isoelectric points have been observed at 0.04 mM and 0.08 mM amphiphiles for mixed IA-16-HSA and PS-16-HSA systems, respectively. It should be noted that experimental data demonstrated that the electrostatic interactions between imidazolium surfactants and HSA are more pronounced. This is reflected in the value of the isoelectric points, as well as in the total charge compensation of the protein.

Fluorescence spectroscopy is an important method for studying surfactant–protein binding. Fluorescence quenching is used as a technique for measuring the binding affinity between proteins and ligands and is expressed as a decrease in the fluorescence quantum yield of fluorophore induced via various intermolecular interactions. HSA fluorescence is caused by tryptophan (Trp), tyrosine (Tyr), and phenylalanine (Phe) residues. However, Phe has a very low quantum yield, and Tyr is almost completely quenched if it is ionized or located near the amino-, carboxylic groups, or Trp 214 residue. Therefore, Trp 214 is largely responsible for the intrinsic fluorescence of HSA, which is located deep in the IIA subdomain [69]. 

**Figure 10:**
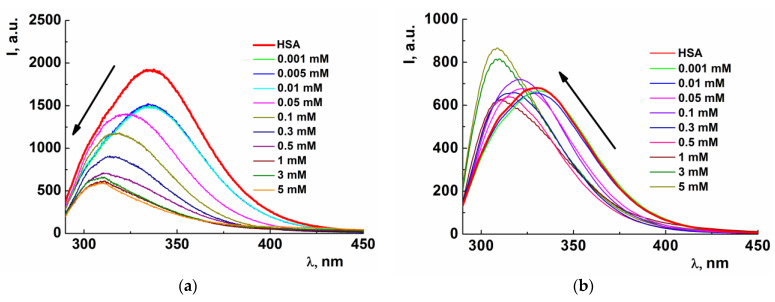
Fluorescence intrinsic spectra of IA-16-HSA (**a**) and PS-16-HSA (**b**) binary systems under various amphiphile concentrations (HSA concentration 5 µM); 25 °C (I-fluorescence intensity).

Figure 10 reveals that the fluorescence spectrum of HSA has an emission peak at 340 nm (λ_ex_ = 280 nm). The addition of cationic surfactants (IA-16 and PS-16) lead to a hypsochromic shift. Such a blue shift in the fluorescence spectra of proteins corresponds to a decrease in the polarity of the microenvironment after the binding of the components, which indicates that the protein chromophore (Trp and Tyr) goes into a more hydrophobic region [44,64,71,72]. However, the addition of IA-16 quenched the HSA fluorescence, while PS-16 increased the fluorescence intensity. Protein fluorescence quenching is a very common phenomenon, which provides evidence for the binding of components [35,44,64,71,72,73]. The protein fluorescence enhancement in the presence of surfactants occurs very rarely and is known as the fluorescence sensitization effect. This effect usually appears when biomacromolecules coexist with some ligands [74,75]. It is interesting to note that the same result was previously obtained in the case of the interaction of a pyrrolidinium surfactant containing carbamate fragment with HSA [47]. 

Fluorescence quenching can occur in two ways: by a collision between the fluorophore and the quencher (dynamic quenching) and by the formation of a complex in the ground state (static quenching). The quenching mechanism can be determined by the value of the bimolecular quenching rate constant, as well as by comparing the values of the Stern–Volmer quenching constant (K_SV_) obtained at different temperatures. During dynamic quenching, the Stern–Volmer constant is maximum, which is associated with a faster diffusion of molecules at the chosen temperature. In the case of static quenching, the Stern–Volmer constant is lower, which is associated with a decrease in hypersensitivity at a given temperature [76,77]. 

**Figure 11:**
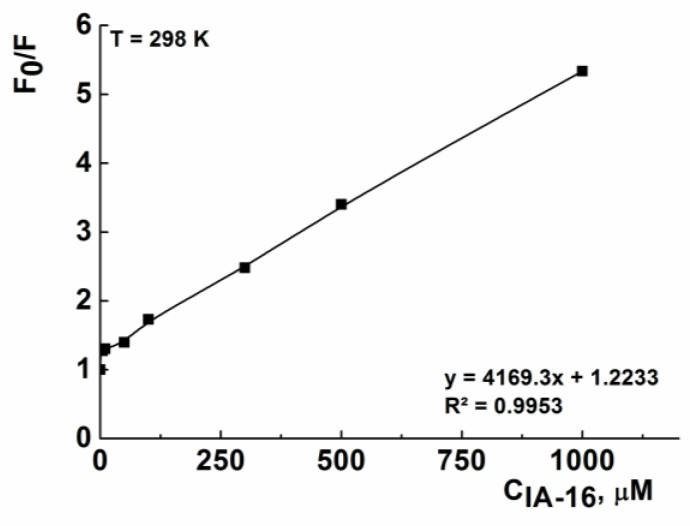
Stern–Volmer plots for the quenching of HSA fluorescence by IA-16 at 298 K. Symbols show the ratio of the fluorescence intensities in the absence and in the presence of the quencher (surfactant) on the surfactant concentration.

Since HSA fluorescence quenching occurred only in the presence of IA-16, several quantitative parameters characterizing the efficiency of component binding were obtained only for the IA-16-HSA system. The K_SV_ was calculated graphically using the Stern–Volmer Equation (1) under various temperatures (Table 3). For example, Figure 11 shows the Stern–Volmer dependences for the IA-16-HSA complex at 25 °C. In turn, the Stern–Volmer constant makes it possible to calculate the bimolecular quenching rate constant (K_q_), which allows one to draw a conclusion about the predominant quenching mechanism in systems. So, the dynamic quenching mechanism prevails if the value of K_q_ is less than 2 × 10^10^ L/mol s and if the static quenching mechanism is more than 2 × 10^10^ L/mol s [44,51,76,77]. Calculated data on various temperatures are given in Table 3. The Stern–Volmer quenching constant increases with increasing temperature for the IA-16-HSA system, which indicates a dynamic quenching mechanism. However, the calculated values of K_q_ are at least 20 times higher than the maximum dynamic constant of bimolecular quenching in an aqueous medium (2 × 10^10^ L/mol s), which indicates a static mechanism of fluorescence quenching. Most likely, the formation of IA-16-HSA complexes is associated with a mixed quenching mechanism, including both the molecular collision and the formation of a non-covalently bound complex due to the adsorption of surfactant molecules on the hydrophobic domains of the protein [34,45,51]. 

A modified version of the Stern–Volmer equation (Equation (1)) can be applied to the calculation of the binding constants of surfactants with HSA (K_a_) and the number of binding sites (n). Table 3 shows that the binding constant decreases with increasing temperature, indicating lower stability of the complex in the ground state at a higher temperature due to the pronounced mobility of the molecules [34,77]. In addition, it should be noted that the K_a_ values obtained for IA-16-HSA suggest a lower binding affinity compared to other strong protein–ligand complexes [34,78]. The n values for IA-16-HSA are at the level of 0.6, which testifies to the presence of one binding site in HSA for surfactants upon their interaction. Further, using the values of the coupling constants of the components K_a_, the thermodynamic parameters of the system were calculated: the change in enthalpy ΔH^0^, entropy ΔS^0^, and Gibbs free energy ΔG^0^ (Equations (3)–(5), Table 3). It is known that, depending on the nature of the change in the thermodynamic functions ΔH^0^ and ΔS^0^, various intermolecular interactions can dominate in the formation of surfactant–HSA complexes [34,51]:

- ΔH^0^ < 0 and ΔS^0^ < 0—hydrogen bonding and Van der Waals interactions predominate;
- ΔH^0^ > 0 and ΔS^0^ > 0—hydrophobic interactions predominate;
- ΔH^0^ < 0 and ΔS^0^ > 0—electrostatic interactions predominate.

Summing up all this, hydrogen bonding and Van der Waals interactions predominate in the IA-16-HSA systems, which is in accordance with the quite low binding constants of surfactants to HSA (K_a_). Negative Gibbs energy demonstrates the thermodynamic advantage of the formation of IA-16-HSA complexes and the spontaneity of the process.

At the next stage, the IA-16 and HSA interactions were studied using three-dimensional fluorescence spectroscopy. A 3D fluorescence is an important method for studying conformational changes in proteins, and it also allows to evaluate the microenvironment around aromatic residues in macromolecules. In this method, the fluorescence intensity is measured for all possible combinations of excitation and emission wavelengths and summarized on a three-dimensional graph [79]. 

**Figure 12:**
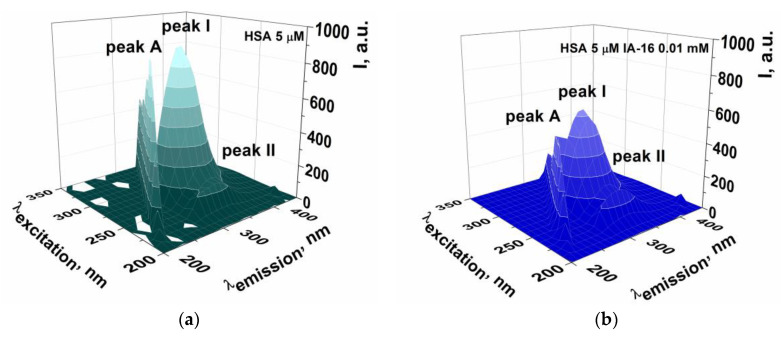

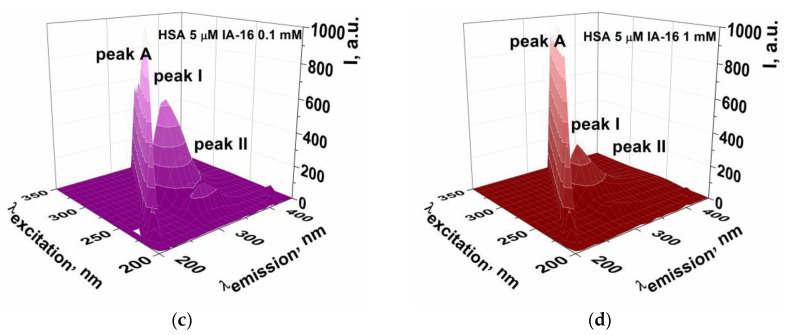
3D fluorescence spectra of pure HSA (**a**) and in the presence of 0.01 mM (**b**), 0.1 mM (**c**), 1 mM (**d**) IA-16.

The 3D fluorescence spectra of pure HSA and in the presence of IA-16 are shown in Figure 12. There are three characteristic peaks in the HSA spectra, namely, peak A, peak I, and peak II. Peak A corresponds to the Rayleigh scattering peak (λ_ex_ = λ_em_). Peak I at λ_ex_ = 280 nm and λ_em_ = 345 nm demonstrates the characteristic HSA fluorescence associated with Tyr and Trp residues, respectively (π→π* transition of aromatic amino acids). Peak II indicates the spectral behavior of the peptide bond skeleton in HSA obtained by the transition of the n–π*electron of the carbonyl group (λ_ex_ = 230 nm, λ_em_ = 345 nm) [47,80]. It can be seen that an increase in the surfactant concentration in the system leads to fluorescence quenching of peak I, as well as to its slight hypsochromic shift (by ~35 nm). This indicates the transition of Tyr and Trp residues into a less polar environment and, consequently, an increase in hydrophobicity around amino acid fragments [79]. The results of 3D fluorescence spectra are in good agreement with the results of conventional fluorescence, which evidence that the interaction of IA-16 and HSA occurs with little effect on protein conformation and the formation of a non-covalently bound complex due to the adsorption of surfactant molecules on the hydrophobic domains of the protein.

### 3.3. Drug Solubilization Study in Surfactant–PE Nanocontainers

Poor water solubility is one of the important limiting factors for drug administration and successful treatment. The most common ways to increase the solubility of drugs are the use of hydrotropes, surfactant-based nanocarriers, and changing environmental conditions, such as temperature, pH, etc. [81]. Micelles attract wide attention, providing multiple increases in solubility of different practically relevant substrates (drugs, food, chemical dyes, cosmetics, pesticides, etc.), which is one of the key properties of amphiphiles from the practical viewpoint. Polymer–colloid complexes are an alternative system for dissolving insoluble substances [13]. However, the solubilization properties of surfactant are more studied than surfactant–PE mixtures. It is most likely that drug solubilization occurs therein at a lower surfactant concentration compared to pure one due to the formation of micellar aggregates at a lower surfactant concentration in the presence of oppositely charged PE. 

**Figure 13:**
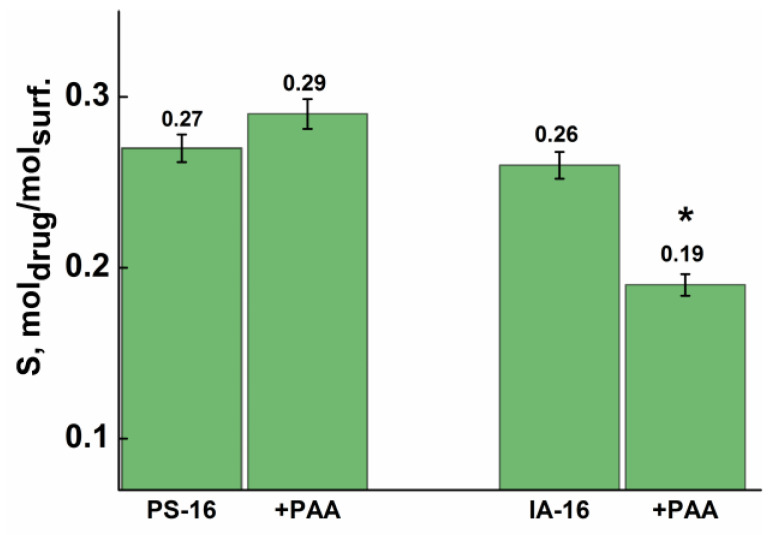
Solubilization capacity of the pure surfactant and mixed surfactant–PAA systems toward Meloxicam. Data are represented as mean ± standard deviation (n = 3); statistically significant differences between PS-16-PAA and IA-16-PAA systems were represented as *—*p* = 0.04.

**Figure 14:**
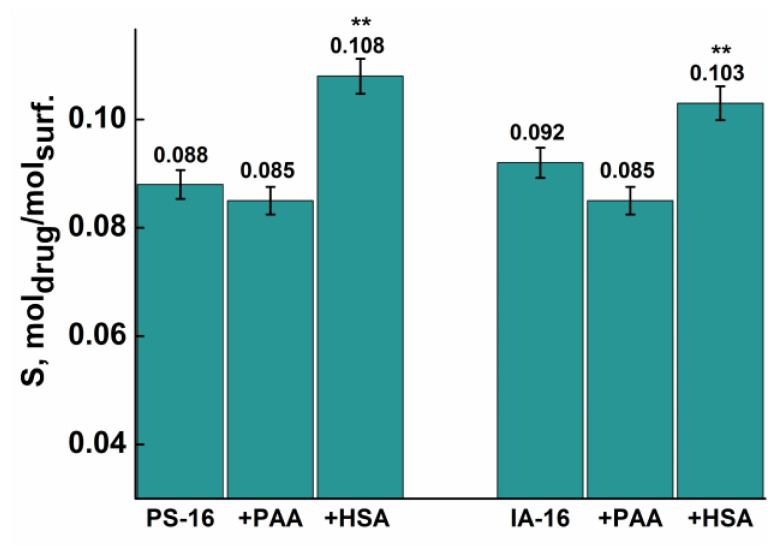
Solubilization capacity of the pure surfactant and mixed surfactant–PE systems toward Warfarin. Data are represented as mean ± standard deviation (n = 3); statistically significant differences in S value between pure surfactant and mixed systems were represented as **—*p* < 0.01.

**Figure 15:**
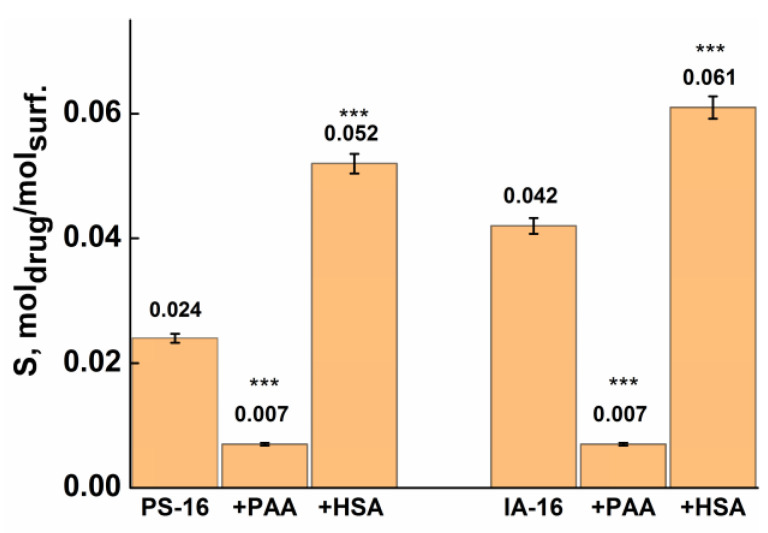
Solubilization capacity of the pure surfactant and mixed surfactant–PE systems toward Amphotericin B. Data are represented as mean ± standard deviation (n = 3); statistically significant differences in S value between pure surfactant and mixed systems were represented as ***—*p* < 0.001.

In accordance with the literature, including our works, the solubilization activity of cationic surfactants depends on the nature of their head group and the type of drug. Moreover, in many cases, the solubilization capacity of cationic amphiphiles with a cyclic head group is higher compared to open head group analogs [81,82]. Therefore, the solubilization ability of mixed surfactant–PE systems toward poorly water-soluble drugs of various therapeutic effects (non-steroidal anti-inflammatory agent (NSAID)-meloxicam (Figure 13), anticoagulant-warfarin (Figure 14), and antifungal antibiotic-amphotericin B (Figure 15)) was investigated.

In accordance with [83], oppositely charged surfactant–PE mixed solutions were incubated with an excess of drugs for 24 h. S is used for quantitative estimation, which is the ratio of the mole of solubilized drug per mole of surfactant. Parameter S of the mixed surfactant–PE systems in relation to the presented drugs was evaluated by UV-vis spectrophotometry. Absorption spectra were recorded (Figures S1, S2, S4–S6 and S8–S10), followed by the construction of concentration dependences of the drug absorbance (Figures S3, S7 and S11). Meloxicam is an NSAID that has analgesic, anti-inflammatory, and antipyretic effects. It is a pH-dependent drug, the solubility of which is determined by the pK_a_ values and the existence ranges of the charged forms of the compound. Therefore, studies were carried out in acetate buffer at pH 4.4; under these conditions, the drug is insoluble in water but soluble in micellar solutions. 

As can be seen from Figure S1, meloxicam is highly soluble in micellar solutions of individual surfactants independent of the structure of the surfactant head group. The peak at 364 nm in the spectra of meloxicam showed maximum absorption and was taken for study (Figures S1 and S2). However, the solubilization capacity (S) of the studied pyrrolidinium surfactant (0.27 mole of meloxicam/mole of PS-16) is much worse compared to PS-16 containing a hydroxyethyl fragment (0.8 mole of meloxicam/mole of PS-16) [83]. It should be noted that solubilization of the drug begins before tensiometric CMC (1 mM) at a concentration of 0.3 mM (Figure S3). It was previously reported that the acetate buffer reduces twice the aggregation thresholds of PS-16 with the hydroxyethyl fragment. The addition of PAA to micellar solutions impairs the solubility of meloxicam. The solubility is three–five times higher in a pure solution compared to mixed systems at comparable surfactant concentrations (Figure S3). It is likely that the polyanion creates a steric barrier and prevents the penetration of the drug into micellar aggregates, despite the fact that the dimensions of polymer–colloidal aggregates are much larger than micelles. At the same time, for the mixed systems containing imidazolium surfactant, the S values are lower by 30% compared to pyrrolidinium surfactant (Figure 13). To estimate the statistical significance, a one-way analysis of variance (ANOVA), where *p* = 0.04.

Next, spectrophotometric studies of the solubility of warfarin in micellar solutions of pyrrolidinium (Figure S4b) and imidazolium surfactants (Figure S4a) in the presence of PAA (Figure S5) and HSA (Figure S6) were carried out. Warfarin is an indirect anticoagulant, a coumarin derivative, which is used for the treatment and prevention of thrombosis and hematogenous embolism. The peak at 307 nm in the spectra of warfarin showed maximum absorbance and was taken to study the solubilization capacity (Figures S4–S6). The studied polyanions have the opposite effect on the solubilization capacity of amphiphiles (Figure 14). PAA additives slightly reduce the S value of the micellar systems, whereas HSA leads to an increase in S by 10–20% with statistically significant differences between the pure surfactant and mixed systems (*p* < 0.01). It is likely that HSA provides favorable hydrophobic binding sites for the drug, as previously was shown for other drugs [84,85]. In addition, binding site 1 of albumins showed a strong affinity for heterocyclic compounds such as warfarin, indomethacin, etc. [86].

Amphotericin B is an effective polyene broad-spectrum antibiotic against fungal diseases with poor solubility in water. Currently, several liposomal forms of amphotericin B have been developed; however, the high cost is the disadvantage of this approach [87]. The application of micellar nanocontainers can significantly reduce the cost and toxicity of the antibiotic. There are three maxima in the absorption spectra of the drug; the peak at 389 nm was chosen to study the solubilization capacity (Figures S8–S10). The most pronounced PE effect on the solubilization ability of amphiphiles is observed in the case of amphotericin B (Figure 15). Modification of nanocontainers with a protein increases the solubilization capacity more than twice, while the synthetic polyanion reduces the drug solubility by six times compared to pure surfactants. This is probably due to the strong affinity of amphotericin B for serum proteins. In work [86], a variety of spectroscopic methods showed a significant decrease in the HSA and BSA intrinsic fluorescence with an increase in the amphotericin concentration, which confirms the effectiveness of the interaction between components. Synthetic polyelectrolytes, specifically PAA, can initiate the formation of mixed surfactant–PE aggregates, similar to a pearl-necklace structure [24], wherein micelles look as strung on a PE chain. In this case, the hydrophobic PE chains probably compete with the hydrophobic drug for the solubilization in micelles. 

## 4. Conclusions

Multifunctional self-assembling systems based on cationic surfactants, synthetic polyanion (polyacrylic acid), and protein (human serum albumin) were formed under varying the structure of the head group of amphiphiles (pyrrolidinium and imidazolium). The properties of the mixed systems have been systematically studied using a variety of modern complementary physicochemical methods. The polyanion additives increased the aggregation activity of the studied amphiphiles in terms of critical aggregation concentrations by two orders of magnitude (from 1 mM to 0.01 mM). The results also revealed a correlation between aggregation thresholds of mixed surfactant–HSA systems and the structure of the surfactant head group; in particular, lower CAC was demonstrated in the case of IA-16. Synthetic macromolecule PAA gives the possibility of obtaining nanoscale mixed micellar aggregates with controlled size and shape depending on the surfactant concentration, while no significant changes in size behavior were observed in the case of HSA. For the mixed system with IA-16, classical quenching of HSA fluorescence was shown, while for the system with PS-16, on the contrary, an unusual increase in fluorescence was observed, which indicates a different binding mechanism of surfactant to the protein. According to the structure–properties relationships revealed, polymer–colloid nanocontainers with controlled parameters and different loading efficiencies of hydrophobic drugs have been developed. It was shown that the solubilization capacity of surfactant–PE mixtures depends on the structure of the surfactant head group and the nature of polyanions. It can be increased more than twice, which was demonstrated on several drugs (meloxicam, warfarin, and amphotericin B). The obtained experimental data extend the understanding of the mechanism of action of polyanions and the biomedical application of amphiphilic compounds.

## Figures and Tables

**Table 1:** CMC/CAC values for pure and mixed surfactant–PAA systems under varying PE concentrations obtained by tensiometry and fluorescence spectroscopy.

Surfactant	C_PAA_, mM	CAC-1/CAC-2 *, mM	
		Tensiometry	Fluorimetry
PS-16	0	1	1
	1	0.060	0.03
	3	0.050	0.02
	5	0.060	0.03
IA-16	0	1	1
	1	0.050/1	0.019
	3	0.080/1	0.015
	5	0.080/1	0.011

* For the case if two breakpoints are observed.

**Table 2:** The hydrodynamic diameters (D_h_), zeta potential (ζ), and polydispersity index (PdI) of PAA-PS-16 and PAA-IA-16 associated under different molar ratio; pH = 4; 25 °C.

No.	C_PAA_, mM	C_surfactant_, mM	D_h_, nm ± SD *	ζ, mV	PdI
	PS-16				
1	1	0.03	90 ± 10	−13 ± 2	0.194 ± 0.102
2		0.05	** 186 ± 2	−10 ± 1	0.071 ± 0.019
3		0.2	60 ± 3	22 ± 3	0.323 ± 0.009
4		0.5	69 ± 2	23 ± 1	0.225 ± 0.012
5	3	0.04	114 ± 12	−22 ± 1	0.298 ± 0.025
6		0.5	88 ± 2	32 ± 5	0.109 ± 0.015
7		1	37 ± 5	37 ± 5	0.219 ± 0.004
8	5	0.02	137 ± 25	−21 ± 1	0.450 ± 0.037
9		0.05	179 ± 3	−17 ± 1	0.430 ± 0.030
10		0.5	98 ± 30	21 ± 1	0.104 ± 0.005
11		1	59 ± 5	43 ± 2	0.212 ± 0.002
	IA-16				
1	1	0.05	98 ± 1	−17 ± 1	0.073 ± 0.021
2		0.5	44 ± 1	−6 ± 1	0.376 ± 0.011
3		1	59 ± 15	50 ± 5	0.244 ± 0.028
4	3	0.02	116 ± 1	−36 ± 1	0.333 ± 0.021
5		0.1	137 ± 5	−22 ± 2	0.148 ± 0.021
6		0.5	94 ± 2	31 ± 1	0.157 ± 0.004
7		1	52 ± 19	61 ± 2	0.323 ± 0.041
8	5	0.05	114 ± 11	−21 ± 1	0.483 ± 0.001
9		0.08	120 ± 13	−19 ± 1	0.325 ± 0.019
10		0.5	135 ± 1	18 ± 1	0.068 ± 0.028
11		1	59 ± 5	54 ± 2	0.122 ± 0.003

* Data are represented as mean ± standard deviation (n = 3). ** PS-16-PAA at 0.05–1 mM, respectively, were chosen for visualization.

**Table 3:** Stern–Volmer constants K_SV_, component binding constants K_a_, bimolecular quenching rate constants K_q_, and the number of HSA and surfactant binding sites n, changes in enthalpy ΔH^0^, entropy ΔS^0^, and Gibbs free energy ΔG^0^ for the systems under varying temperatures.

T, K	K_SV_·10^3^, L/mol	Kq·10^10^, L/mol·s	K_a_·10^3^, L/mol	n	ΔH^0^, kJ/mol	ΔS^0^, J/mol·K	ΔG^0^, kJ/mol
298	4.169	42.54	0.151	0.6	−21.32	−29.93	−12.40
303	4.417	44.17	0.126	0.6			−12.25
308	4.443	44.43	0.110	0.6			−12.10
313	4.547	45.47	0.100	0.6			−11.95

## Data Availability

Not applicable.

## Supplementary Materials

The following supporting information can be downloaded at: https://www.mdpi.com/article/10.3390/nano13061072/s1, Figure S1: Absorbance spectra of Meloxicam in the IA-16 (a) and PS-16 (b) systems under varying surfactant concentration; acetic buffer pH = 4.4; 25 °C.; Figure S2: Absorbance spectra of Meloxicam in the oppositely charged IA-16-PAA (a) and PS-16-PAA (b) systems under varying surfactant concentration; acetic buffer pH = 4.4; 1 mM PAA; 25 °C; Figure S3: Meloxicam absorbance at 305 nm wavelength upon amphiphile concentration plot for pure surfactant and surfactant–PAA (b) systems; l = 0.5 cm; 25 °C; Figure S4: Absorbance spectra of Warfarin in the IA-16 (a) and PS-16 (b) aqueous solutions under varying surfactant concentration; 25 °C; Figure S5: Absorbance spectra of Warfarin in the oppositely charged IA-16-PAA (a) and PS-16-PAA (b) systems under varying surfactant concentration; 1 mM PAA; l = 0.5 cm; 25 °C; Figure S6: Absorbance spectra of Warfarin in the oppositely charged PS-16-HSA (a) and IA-16-HSA (b) systems under varying surfactant concentration; 0.5 mM HSA; l = 0.5 cm; 25 °C; Figure S7: Warfarin absorbance at 305 nm wavelength versus amphiphile concentration plot for the pure surfactant, surfactant–HSA systems (a) and surfactant–PAA (b) systems; l = 0.5 cm; 25 °C; Figure S8: Absorbance spectra of Amphotericin B in the IA-16 (a) and PS-16 (b) aqueous solutions under varying surfactant concentration; 25 °C; Figure S9: Absorbance spectra of Amphotericin B in the oppositely charged IA-16-PAA (a) and PS-16-PAA (b) systems under varying surfactant concentration; 1 mM PAA; l = 0.5 cm; 25 °C; Figure S10: Absorbance spectra of Amphotericin B in the oppositely charged PS-16-HSA (a) and IA-16-HSA (b) systems under varying surfactant concentration; 0.5 mM HSA; l = 0.5 cm; 25 °C; Figure S11: Amphotericin B absorbance at 385 nm wavelength upon amphiphile concentration plot for pure surfactant, surfactant–HSA (a) and surfactant–PAA (b) systems; l = 0.5 cm; 25 °C; Table S1: The hydrodynamic diameters (D_h_), zeta potential (ζ) and polydispersity index (PdI) of PAA-PS-16 and PAA-IA-16 associates under different molar ratio; pH = 4; 25 °C; *p* < 0.05 means statistically significant difference values, *p* > 0.05 no significance.
