# Supplementary material for: Role of Polyanions and Surfactant Head Group in the Formation of Polymer–Colloid Nanocontainers
Source: Nanomaterials (Basel). 2023 Mar 16;13(6):1072. doi: 10.3390/nano13061072 (PMC10056398; doi:10.3390/nano13061072)
Supplement: Supplementary file 1 [file nanomaterials-13-01072-s001.zip › nanomaterials-2244006-supplementary.pdf]

nanomaterials

MDPI

Supplementary Materials

Role of Polyanions and Surfactant Head Group in the Formation of Polymer–Colloid Nanocontainers

Elmira A. Vasilieva *, Darya A. Kuznetsova, Farida G. Valeeva, Denis M. Kuznetsov and Lucia Ya. Zakharova

Arbuzov Institute of Organic and Physical Chemistry, FRC Kazan Scientific Center, Russian Academy of Sciences, Arbuzov Str. 8, 420088 Kazan, Russia
* Correspondence: vasilevaelmira@mail.ru

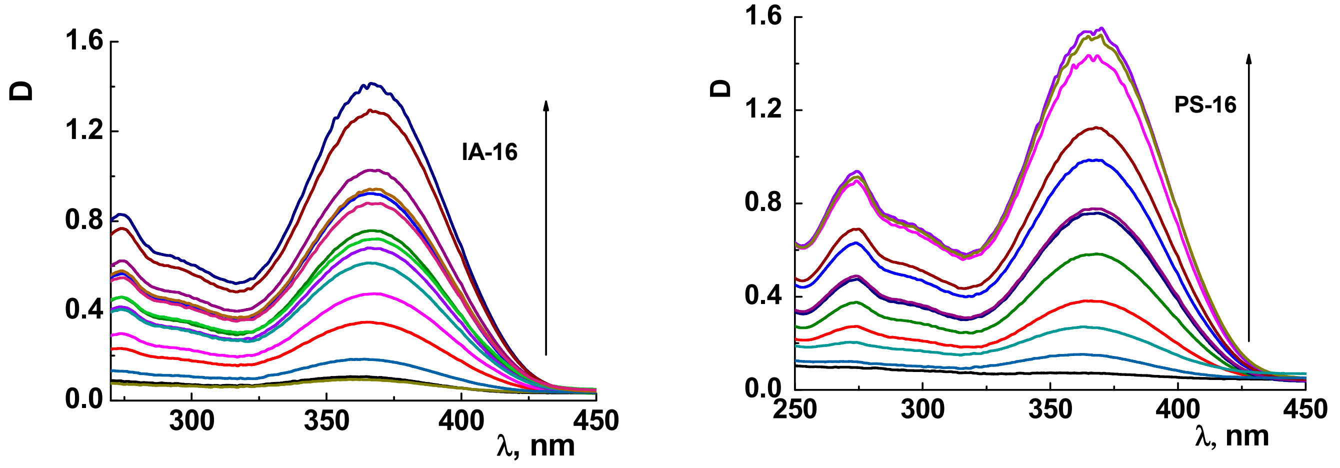

(a) (b)

Figure S1. Absorbance spectra of Meloxicam in the IA-16 (a) and PS-16 (b) systems under varying surfactant concentration; acetic buffer pH=4.4; 25°C.

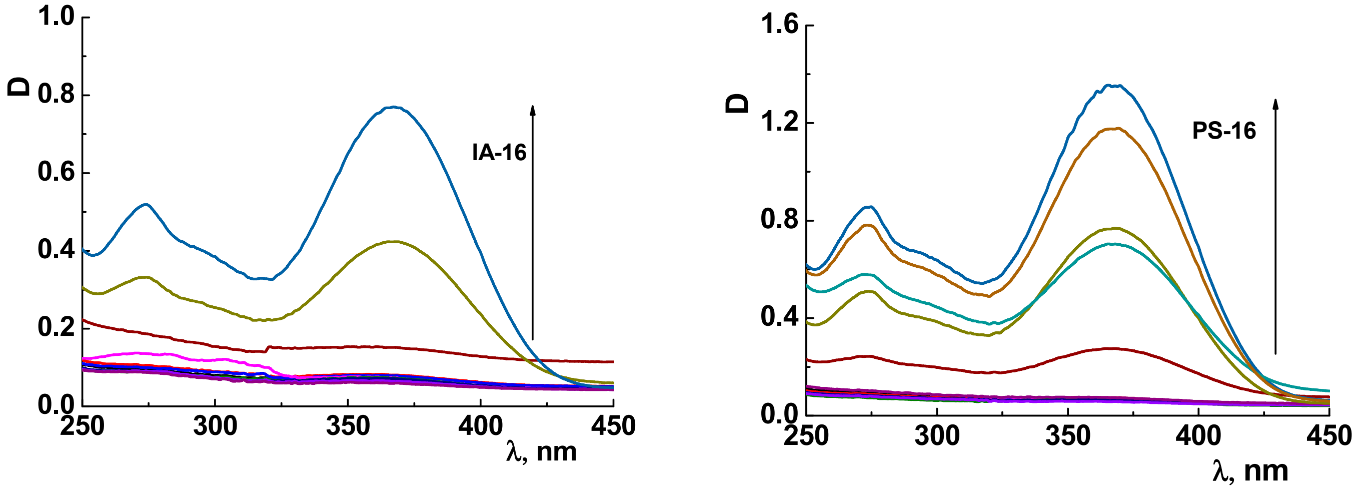

(a) (b)

Figure S2. Absorbance spectra of Meloxicam in the oppositely charged IA-16-PAA (a) and PS-16-PAA (b) systems under varying surfactant concentration; acetic buffer pH=4.4; 1 mM PAA; 25°C.

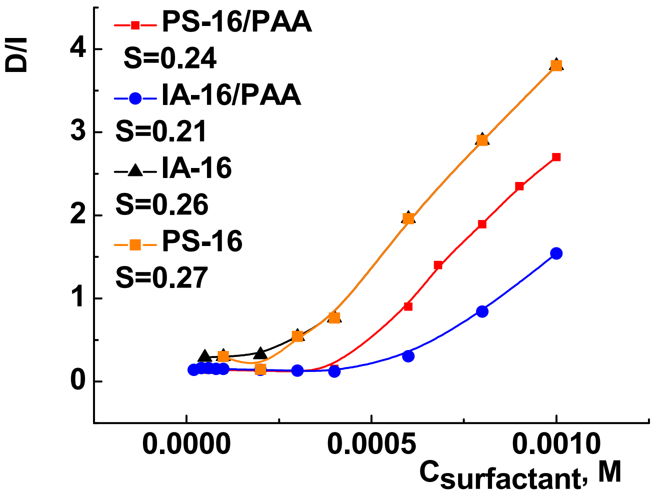

Figure S3. Meloxicam absorbance plot at 305 nm wavelength upon amphiphile concentration plot for pure surfactant and surfactant-PAA (b) systems; l=0.5 cm; 25°C.

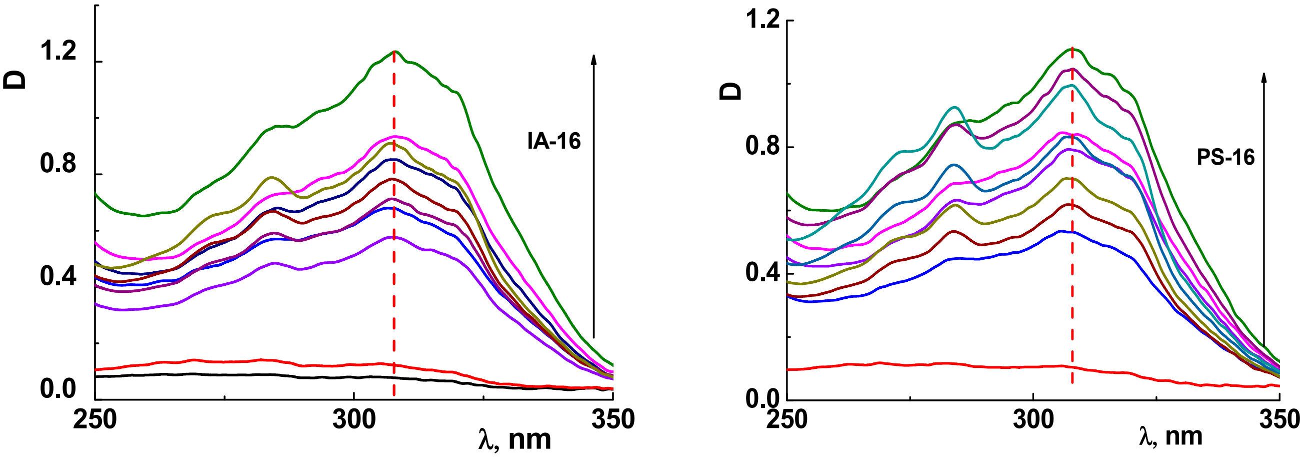

(a) (b)

Figure S4. Absorbance spectra of Warfarin in the IA-16 (a) and PS-16 (b) aqueous solutions under varying surfactant concentration; 25°C.

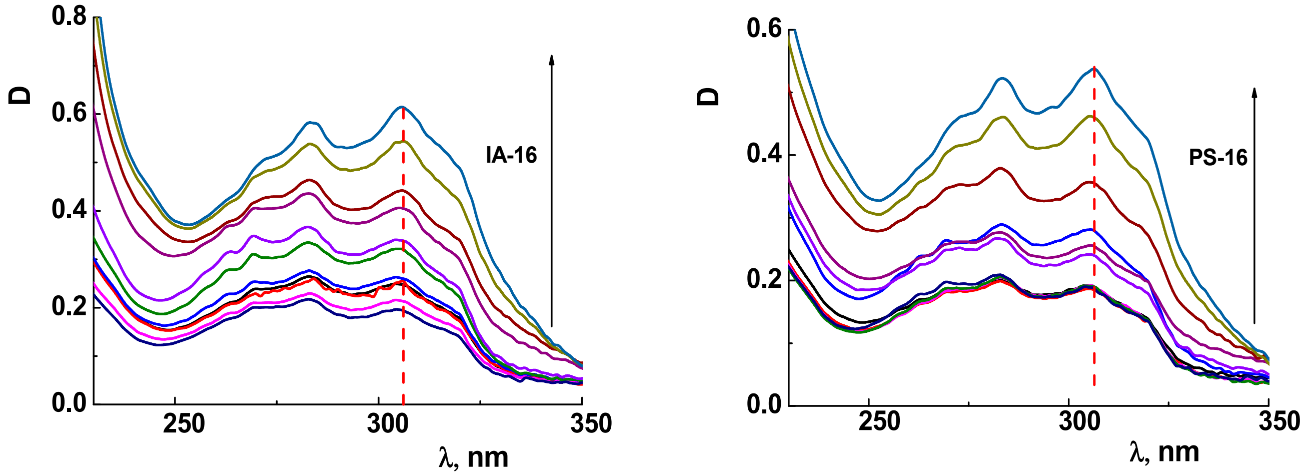

(a) (b)

Figure S5. Absorbance spectra of Warfarin in the oppositely charged IA-16-PAA (a) and PS-16-PAA (b) systems under varying surfactant concentration; 1 mM PAA; l=0.5 cm; 25°C.

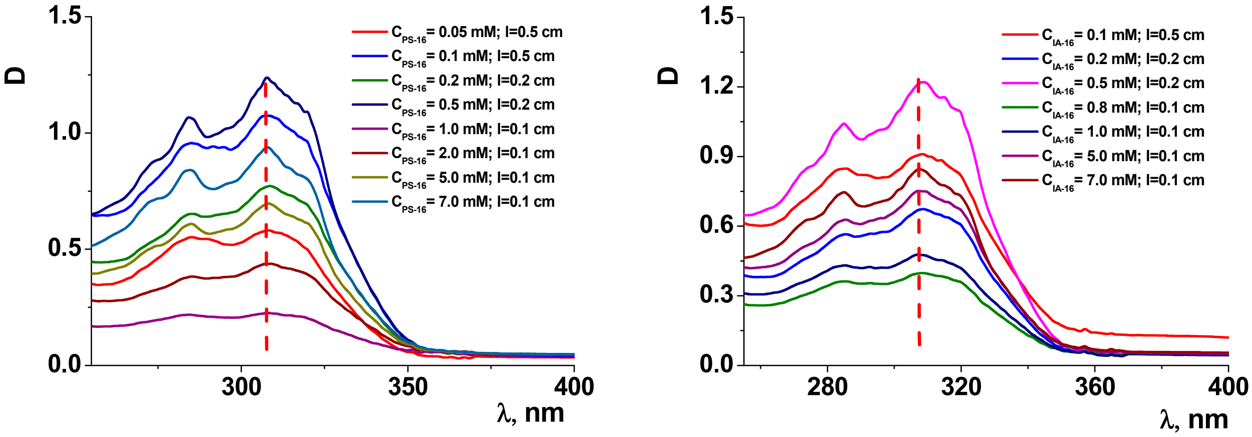

(a) (b)

Figure S6. Absorbance spectra of Warfarin in the oppositely charged PS-16-HSA (a) and IA-16-HSA (b) systems under varying surfactant concentration; 0.5 mM HSA; l=0.5 cm; 25°C.

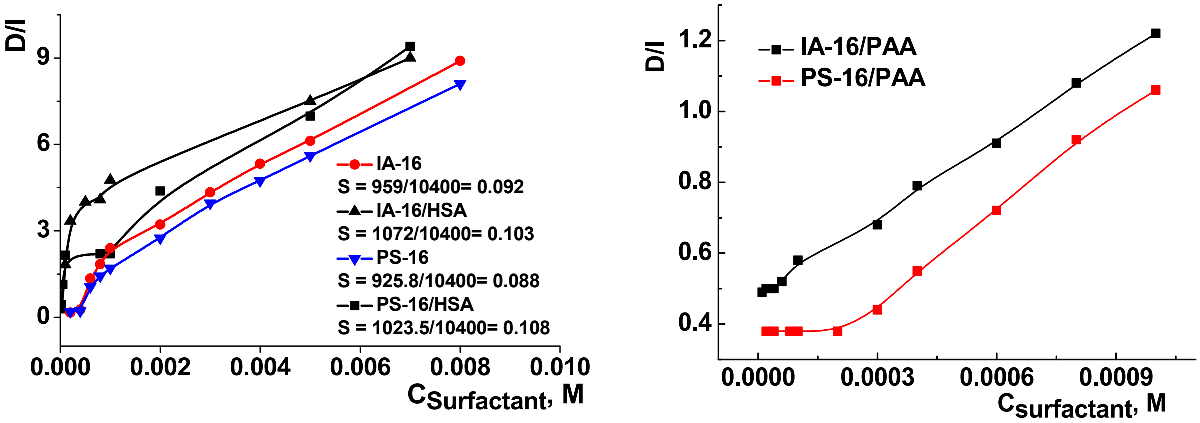

(a) (b)

Figure S7. Warfarin absorbance plot at 305 nm wavelength versus amphiphile concentration for the pure surfactant, surfactant-HSA systems (a) and surfactant-PAA (b) systems; l=0.5 cm; 25°C.

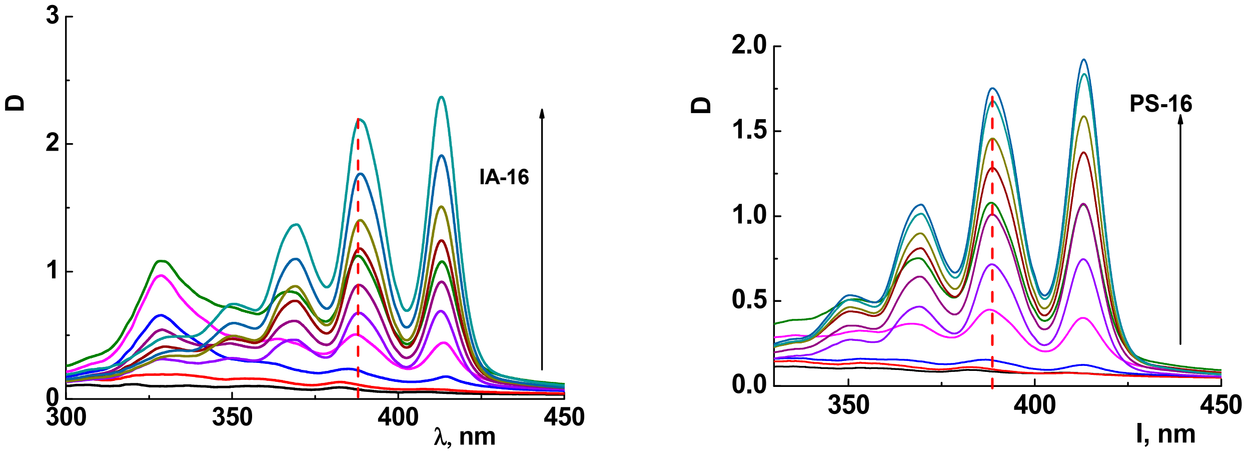

(a) (b)

Figure S8. Absorbance spectra of Amphotericin B in the IA-16 (a) and PS-16 (b) aqueous solutions under varying surfactant concentration; 25°C.

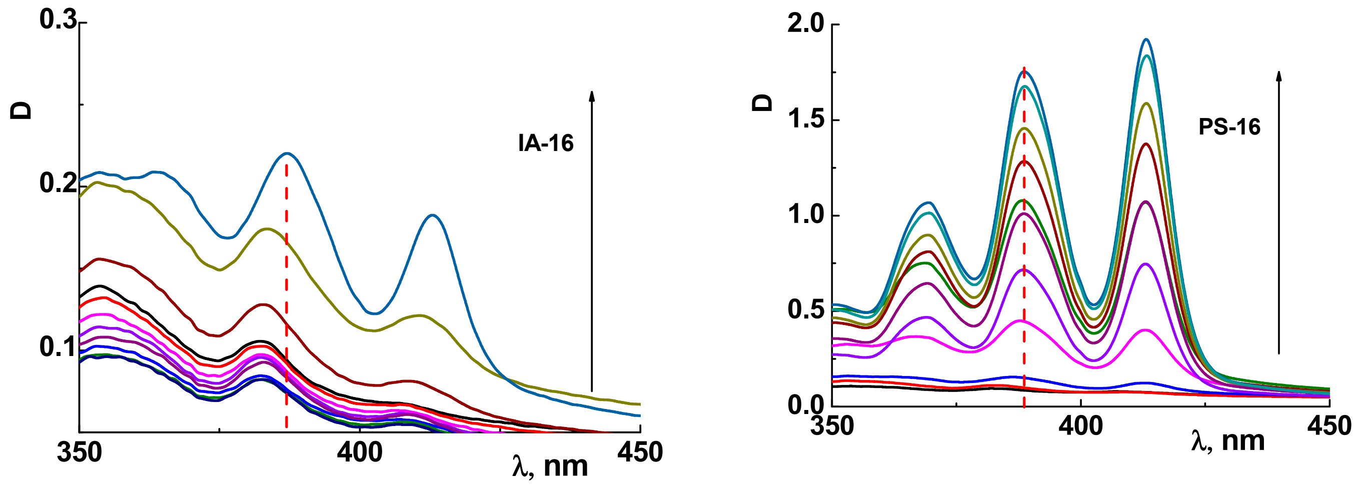

(a) (b)

Figure S9. Absorbance spectra of Amphotericin B in the oppositely charged IA-16-PAA (a) and PS-16-PAA (b) systems under varying surfactant concentration; 1 mM PAA; l=0.5 cm; 25°C.

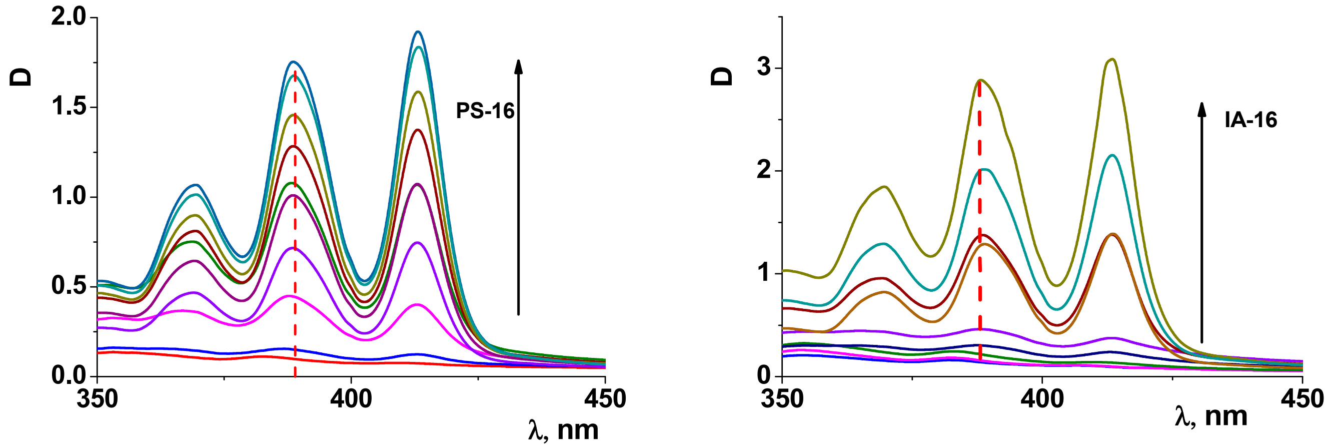

(a) (b)

Figure S10. Absorbance spectra of Amphotericin B in the oppositely charged PS-16-HSA (a) and IA-16-HSA (b) systems under varying surfactant concentration; 0.5 mM HSA; l=0.5 cm; 25°C.

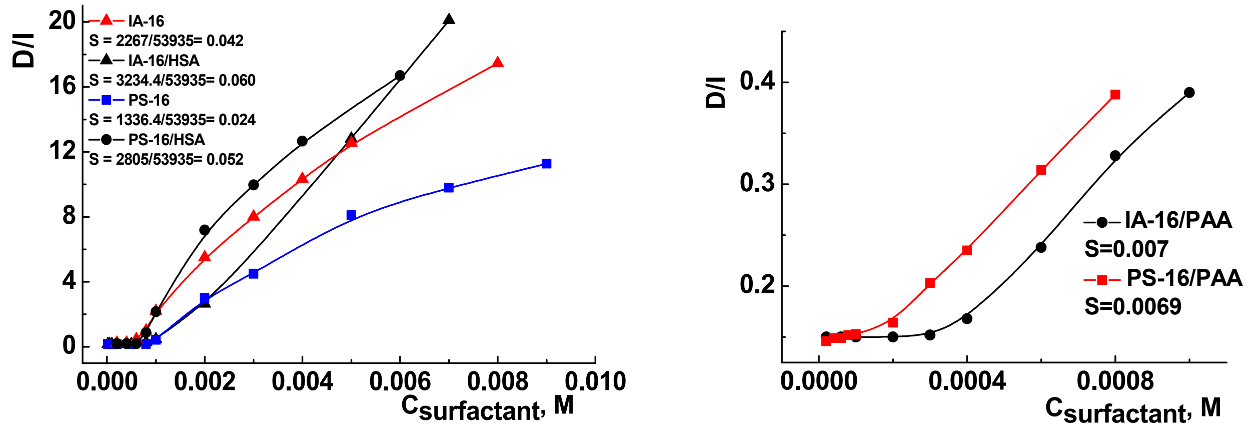

(a) (b)

Figure S11. Amphotericin B absorbance plot at 385 nm wavelength upon amphiphile concentration for pure surfactant, surfactant-HSA (a) and surfactant-PAA (b) systems; l=0.5 cm; 25°C.

Table S1. The hydrodynamic diameter (D_h), zeta potential (ζ) and polydispersity index (PdI) of PAA-PS-16 and PAA-IA-16 associates under different molar ratio; pH=4; 25°C; p<0.05 means statistically significant difference values, p>0.05 no significance.

№	C_{PAA}, mM	$C_{surfactant}$, mM	D_h, nm (number)	ζ, mV	PdI
			PS-16		
1	1	0.03	*90±10	-13±2	0.194±0.102
2		0.05	*186±2	-10±1	0.071±0.019
3		0.2	*60±3	22±3	0.323±0.009
4		0.5	69±2	23±1	0.225±0.012
D_h: p=2E-8 for №1,2; p=7E-4 for №3,1; p=0.001 for №3,2; 4,2; p=0.006 for №4,1; p>0.05 for №4,3.					
5	3	0.04	*114±15	-22±1	0.298±0.025
6		0.5	*88±2	32±5	0.109±0.015
7		1	*37±5	37±5	0.219±0.004
D_h: p=0.02 for №6,5; p=1E-4 for №7,5; p=0.001 for №7,6;					
8	5	0.02	137±25	-21±1	0.450±0.037
9		0.05	179±3	-17±1	0.430±0.030
10		0.5	98±30	21±1	0.104±0.005
11		1	59±5	43±2	0.212±0.002
D_h: p>0.05 for №8,9; 10,8; p=0.005 for №10,9; №11,8; p=3E-4 for №11,9;					
			IA-16		
1	1	0.05	98±1	-17±1	0.073±0.021
2		0.5	44±1	-6±1	0.376±0.011
3		1	59±15	50±5	0.244±0.028
D_h: p=0.001 for №3,1; p=2E-4 for №2,1; p>0.05 for №3,2;					
4	3	0.02	116±1	-36±1	0.333±0.021
5		0.1	137±5	-22±2	0.148±0.021
6		0.5	94±2	31±1	0.157±0.004
7		1	52±19	61±2	0.323±0.041
D_h: p=0.02 for №5,4; 6,4; p=4E-4 for №6,5; 7,6; p=2E-5 for №7,4; 7,5;					
8	5	0.05	114±11	-21±1	0.483±0.001
9		0.08	120±13	-19±1	0.325±0.019
10		0.5	135±1	18±1	0.068±0.028
11		1	59±5	54±2	0.122±0.003
D_h: p>0.05 for №9,8; 10,8; 10,9; p=4E-4 for №11,9; №11,8; p=2E-5 for №11,10;					
